# STK1p as a prognostic biomarker for overall survival in non-small-cell lung carcinoma, based on real-world data

**DOI:** 10.2144/fsoa-2020-0130

**Published:** 2020-11-23

**Authors:** Zhenxin Wang, Guoqing Zhang, Zhongcheng Li, Jin Li, Hongbo Ma, Ailian Hei, Shunchang Jiao, Yi Hu, Shengjie Sun, Liangliang Wu, Ji Zhou, Yu Wang, Ellen He, Sven Skog

**Affiliations:** 1 Department of Oncology, Affiliated Hospital of Suzhou University, Shizi Street 188, Gusu District, Suzhou, China; 2 Department of Oncology, Peking 301 Chinese PLA General Hospital, Fuxing Road 28, Haidian District, Beijing 100853, China; 3 Department of Thoracic Surgery, Affiliated Hospital of Suzhou University, Shizi Street 188, Gusu District, Suzhou, China; 4 Department of Medicine, Shenzhen SSTK Precision Medicine Institute, A301, Building 1, 1301-76 Guanguang Road, Longhua District, Shenzhen, China; 5 Health Management Center of PLA 910 Hospital, Quanzhou 362000, Fujian, China

**Keywords:** lung cancer, non-small-cell lung cancer (NSCLC), radical resection (RR), serum thymidine kinase 1 protein (STK1p), survival, thymidine kinase 1 (TK1)

## Abstract

**Aim:**

A prospective investigation of serum thymidine kinase 1 concentration (STK1p) was performed to evaluate its prognostic value in patients with non-small-cell lung carcinoma (NSCLCs).

**Patients & methods:**

The STK1p values of 127 patients were determined by an enhanced chemiluminescent dot blot assay. The patients were recruited from March 2011 to December 2017.

**Results:**

Kaplan–Meier plot showed that patients with elevated STK1p values had worse overall survival (OS), especially patients of early/middle stages. Multi-variable COX regression showed that STK1p value and combined treatment surgery + chemotherapy were independent prognostic factors for favorable OS.

**Conclusion:**

STK1p is helpful in predicting OS of early/middle stages (I–IIIA) NSCLCs patients following a nonrandomized individual adapted treatment, but is may be not recommended in advanced stages (IIIB + IV) of NSCLCs.

## Lay abstract

Lung cancer is one of the most common types of tumors, with a high mortality rate. We investigate if thymidine kinase 1 in serum (STK1p) is a reliable prognostic marker for survival in non-small-cell lung carcinoma. We recruited 127 patients in this study. STK1p level was determined using a high-sensitive chemiluminescent dot blot assay. Patients with elevated STK1p values had worse overall survival, especially patients in the early/middle cancer stages. Analysis showed that STK1p is an independent prognostic factors for overall survival. We concluded that STK1p is helpful predicting the efficacy of treatment in non-small-cell lung carcinoma for those in the early/middle stages.

Lung cancer is one of the most common types of tumors, with a high mortality rate [1]. According to Global cancer statistics (2018), 2,093,876 new lung cancer cases and 1,761,007 deaths have been reported. Lung cancer can be categorized as 85% non-small-cell lung cancer (NSCLC) and 15% small-cell lung cancer (SCLC). The 5-year survival rate for NSCLC patients remains very low (about 16% at 5 years) [2,3]. Owing to the lack of typical symptoms and ineffective diagnostic and prognostic methods, the recurrence rates postsurgery remain high due to the presence of micrometastatic disease at the time of operation. Two large randomized controlled trials showed that early detection with low-dose computed tomography and early-stage treatment can reduce mortality [4]. A series of tumor-related serum/plasma biomarkers for identifying early stages of lung cancer in routine setting are in use, such as CEA, CYFRA21-1 [5] or CY211 [6]. Those tumor-related markers, however, may not directly reflect tumor proliferating rate [7–9]. In lung cancer, receiver operation characteristic (ROC) statistical analysis has also demonstrated that the sensitivity and specificity of STK1p value were significantly higher when combined with CEA, CYFRA21-1 and NSE than that of each biomarker alone (p < 0.0001) [10].

Thymidine kinase 1 (TK1) expression was found to be closely related to cell cycle since the 1950s [11]. TK1 is a precise proliferating biomarker, converting deoxythymidine (dTdR) to deoxythymidine monophosphate (dTMP), followed by incorporation into DNA [12–17]. The levels of serological TK activity [17,18] and serological TK1 protein concentration (STK1p) [16,19–24] in cancer patients are proportional to tumor proliferation. A meta-analysis of 1,694 lung cancer patients demonstrated that the half-life of STK1p is a valuable tool for monitoring surgical response [25]. STK1p is also a potential proliferating biomarker in healthy screening for detection of invisible tumors, as well as for discovery of early-stage tumors [8,9,16,26–28]. In addition, randomized end trials following the reporting recommendations for tumor marker prognostic studies (REMARK) showed that STK1p in multi-variate COX proportional hazards analysis was an independent predictor of recurrence in locoregional breast cancer [27], and in non-Hodgkin’s lymphoma [28]. Similar results were obtained for serum TK activity [29].

In real clinical practice, most cancer patients are faced with a number of uncertainties, which makes it difficult to follow recommendations based on randomized clinical trials. Thus, it is often necessary to modify the treatment plan based on clinical trials in order to guarantee the patient’s survival efficiency. The concept of real-world data (RWD) was suggested by Kaplan *et al.* [30] when analyzing a nonrandomized trial of Ramipril in patients with hypertension. Since then, the concept of RWD has been practiced, using clinical data even from nonrandomized studies [31]. The US FDA has now developed recommendations for such situations [32]. In randomized clinical trials on breast cancer patients, we found that STK1p was an independent prognostic factor for recurrence, based on COX analysis [22]. In a similar retrospective nonrandomized investigation of patients with locoregional breast cancer after adjuvant chemotherapy programs based on the concept of RWD, the STK1p was also found to be an independent prognostic factor for both recurrence and survival [24]. This convinced us that RWD is possible to use for early assessment of prognosis in nonrandomized trails. However, it is important to remember that when using the RWD concept it is necessary to give as much detail as possible as to the clinical data of the patients, as in a randomized trial.

In this prospective study on NSCLCs patients based on the nonrandomized RWD concept, we hypothesized that STK1p can be used as a prognostic factor in early clinical stages (I–IIIA) of individually adapted treatment of NSCLC patients.

## Methods

### Patient selection

**Figure 1.**
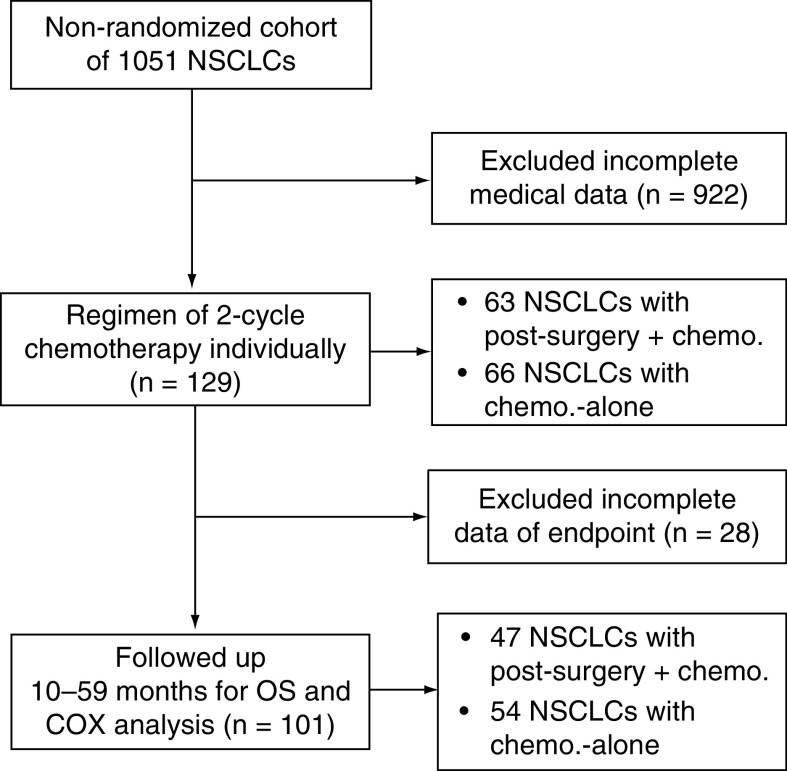
Flow chart of data selection for the investigation of real-world data. Chemo.: Chemotherapy; NSCLC: Non-small-cell lung cancer; OS: Overall survival.

This study was based on a nonrandomized cohort of 1,051 NSCLC patients recruited at the Department of Oncology, Affiliated Hospital of Suzhou University, JiangSu, China, and at the Department of Oncology of Chinese PLA General Hospital, Beijing, China. All of those patients were treated (see below). Of this cohort, 129 patients with survival data were selected (Figure 1). They included:

Affiliated Hospital of Suzhou University: stage I–IIB, n = 25; stage IIIA, n = 9; stage IIIB–IV, n = 51; unclear stages, n = 8; recruited from March 2011 to December 2017.

Department of Oncology of Chinese PLA General Hospital: stage IIIB–IV, n = 36; recruited from February 2014 to December 2017.

These latter patients were validated as wild type for EGFR. All medical information are summarized in Table 1, including age, sex, clinical stages, pathological type (adenocarcinoma [AC], squamous cell carcinoma [SCC]) and type of treatments. Of the 129 patients, 33 were smokers and 55 patients were nonsmokers.

**Table 1.** Clinical information.

Type	Patients (n = 129)	Follow-up, patients (n = 101)
STK1p, preindividual chemo.	129	101
STK1p, two-cycle postindividual chemo.	129	101
Mean age	61.80 ± 9.37 (29–76 years)	61.70 ± 8.17 (29–76 years)
Gender		
Men	96	68
Female	33	33
Histological type		
AC	87	70
SCC	38	28
Alveolar cell carcinoma	1	1
missing	3	1
Clinical stage		
Early/middle: I–IIIA	34	24
I	IA (5), IB (9)	IA (5), IB (4)
II	IIA (8), IIB (3)	IIA (6), IIB (3)
IIIA	IIIA (9)	IIIA (6)
Advanced: IIIB–IV	87	71
IIIB	IIIB (9)	IIIB (9)
IV	IV (78)	IV (62)
Unclear	8	6
Treatment regimen		
Surgery + chemo	63	47
Stage I	IA (5), IB (10)	IA (5), IB (4)
Stage II	IIA (8), IIB (3)	IIA (6), IIB (3)
Stage IIIA	IIIA (9)	IIIA (6)
Stage IIIB + IV	IIIB + IV (20)	IIIB + IV (18)
Unclear stage	8	
Chemo-alone	66	54
IIIB	IIIB (9)	IIIB (9)
IV	IV (57)	IV (45)

AC: Adenocarcinoma; Chemo.: Chemotherapy; SCC: Squamous cell carcinoma; STK1p: Serum thymidine kinase 1 protein.

Serum samples were collected prechemotherapy and after two cycles of chemotherapy treatments (Table 1, treatment details see below).

Serum samples of healthy controls (n = 400, mean age 60.6 ± 7.8, range 51–87 years) were collected between January 2016 and December 2017 at the Health Management Centre of PLA 180 Hospital, Quanzhou, Fujian, China. The healthy controls had no evidence of contagious or cancerous disease and sample collection was approved by the ethics committee of Fujun 180 Hospital, Quanzhou, China (no. LL2009003).

### STK1p in relation to cycles of chemotherapy

In order to find the suitable cycles of chemotherapy when STK1p expression significantly correlated to objective response (OR) including complete response (CR) + partial response (PR); or to nonresponse (NR) including stable disease (SD) + progressive disease (PD) based on the Response Evaluation Criteria in Solid Tumours (RECIST) guideline (version 1.1), we performed a pretest on 88 NSCLC patients (stages I–III) before and after two, four, six and eight cycles of chemotherapy, at the Oncology Department of the First Affiliated Hospital of Suzhou University. We used CYFRA21-1 as a control of the correlation between the RECIST and STK1p. We found that the STK1p and CYFRA21-1 values correlated significantly to the OR value after two-cycle individual-chemotherapy (p = 0.014 and 0.013, respectively) but not after four, six or eight individual-chemotherapy cycles (p > 0.05, date not shown). Therefore, STK1p values were determined during 1 week between the second and the third cycle as the best time to evaluate the prognosis by STK1p. The second cycle of chemotherapy as the best time for evaluation of STK1p for prognosis has been suggested by previous studies, for example, in NSCLC [29] and in breast carcinoma [33].

### Treatment

Treatments were done according to the National Comprehensive Cancer Network (NCCN) Guidelines for patients of Non-Small Cell Lung Cancer, version 2.2010 (www.nccn.org). However, each patient received an individualized chemotherapy program depending on the individual physiological and clinical symptoms, including TNM clinical staging and pathological type of the tumor. We divided patients into two groups of clinical stages, that is, early (IA–IIB)/middle (IIIA) stages and advanced stages (IIIB–IV). Briefly:

- All patients with early/middle stages received radical resection (RR), while only 20 patients of advanced clinical stages (IIIB–IV) received local surgery at different times before chemotherapy.
- Sixty-six patients with advanced clinical stages (IIIB–IV) received only chemotherapy (Table 1).
- According to the instruction of the NCCN guidelines, the patients were treated with individualized chemotherapy, with different cytotoxic agents, given intravenous injection for two cycles (3 weeks/one cycle) and oral administration (2–3 weeks/one cycle). These included docetaxel + cisplatin, gemcitabine + cisplatin, pemetrexed disodium + cisplatin, pemetrexed disodium + nedaplatin, pemetrexed disodium + carboplatin, gemcitabine + nedaplatin, doxorubicin + carboplatin, vinorelbine + nedaplatin.
- The treatment efficacy was evaluated by comparing the STK1p values before chemotherapy and after two cycles chemotherapy. STK1p values of healthy peoples were used as controls. The chemotherapy effect was also evaluated according to WHO RECIST by using advanced imaging, such as CT, abdominal ultrasound and MRI.

### Follow-up

The end point of the 101 NSCLC patients was overall survival (OS) during a follow-up period of 10–59 months. The criteria included were STK1p levels, age, sex, clinical stages, pathological types (AC and SCC) and RECIST. The criteria excluded were patient’s ID, phone number, home address, date when visiting/leaving the hospital, routine blood and urine test results. The patients or their families were contacted by phone every month to check their status. The survival assessment was performed only in 101 patients, due to limited access to 28 patients once they left the hospital.

### STK1p assay

STK1p concentrations were determined using a commercial kit (cat. no. 24/48T) based on an enhanced chemiluminescent dot blot assay as described by the manufacturer Sino-Swed Tongkang Bio-Tech, Inc., Shenzhen, China (www.sstkbiotech.com). The serum samples were taken on the day before the start of the chemotherapy. The serum sample after the second cycle of treatment was taken at day 6 of interval time within 1 week after the second cycle treatment. The collection of serum was performed between 7.30 and 10.00 a.m. after the patients had fasted for 12–14 h. The samples were analyzed within 3 h of whole blood centrifugation (400×*g*, 22–25°C, 8–10 min). If not analyzed immediately, the serum samples were stored at -20°C for a maximum of 4 weeks. While the serum samples were analyzed within 4 weeks, the serum could be stored for ≥1 year at -20°C and still maintain TK1 in good condition. Samples comprising 3 μl serum were directly applied to nitrocellulose membranes in duplicate. Serum samples were then probed with chicken antihuman TK1 IgY polyclonal antibody (dilution 1:500) raised against a peptide (residue 195–225 of human TK1, amino acid sequence: GQPAG PDNKE NCPVP GKPGE AVAAR KLFAPQ; Multiple Peptide Systems (CA, USA). The TK1 calibrators were dotted onto membranes at different concentrations (2.2, 6.6 and 20 pM) as an extrapolated standard. The intensity of spots on the membrane was determined using a *CIS*-l Imaging System (Sino-Swed Tongkang Bio-Tech Inc., Shenzhen, China). The STK1p value of each spot was calculated and expressed as pM, based on the TK1 calibrators.

### Statistical analysis

The data analysis was performed with the statistical program SPSS 25.0 (IBM Corp., NY, USA). The ROC analysis was performed to decide the optimal cut-off value of STK1p. Kaplan–Meier methodology was applied for OS analysis. Uni- and multi-variable COX regression analyses were utilized to recognize risk factors associated with OS. STK1p among the different patient groups after two cycles of chemotherapy used paired-samples *t*-test and one-way ANOVA for comparison of patients and healthy control groups, respectively. Chi-square test was used for the comparison of STK1p in relation to different clinical characteristics. p < 0.05 was considered to indicate a statistically significant value.

## Results

### STK1p levels before & after two-cycle chemotherapy

The patients were divided into five groups (Table 2):

- The original total number of 129 patients (clinical stages IA–IV, Table 2A).
- Follow-up patients from total clinical stages (IA–IV, n = 101, Table 2B).
- Early/middle stages (I–IIIA, n = 34, Table 2C).
- Advanced stages (IIIB + IV, n = 71, Table 2D).
- Controls (n = 400, Table 2E).

**Table 2.** Mean STK1p values in relation with clinical stages before and after two-cycle chemotherapy. (A) Total patients of stages IA–IV (n = 129); (B) followed-up patients (n = 101), clinical stages IA–IV; (C) early/middle stages (IA–IIIA); (D) advantage stages (IIIB–IV); (E) healthy persons as controls.

Group	Samples (n)	STK1p (pM)	p-value	t-value
A. Clinical stage IA–IV	129			
1. Prechemotherapy		1.07 ± 1.54		
2. Post-chemotherapy		0.72 ± 0.95	0.025	2.274
B. Clinical stage IA–IV follow-up	101			
1. Prechemotherapy		1.08 ± 1.58		
2. Post-chemotherapy		0.75 ± 1.03	0.022	2.33
C. Clinical stage I–IIIA	34			
1. Prechemotherapy		0.698 ± 0.767		
2. Post-chemotherapy		0.566 ± 0.774	0.366	0.917
D. Clinical stage IIIB–IV	71			
1. Prechemotherapy		1.148 ± 1.70		
2. Post-chemotherapy		0.752 ± 1.03	0.016	2.4698
E. Controls	400	0.38 ± 0.31	<0.001 (E vs A, B, C or D)	

STK1p: Serum thymidine kinase 1 protein.

In general, pretreatment mean value of STK1p for malignant patients is above 2.0 pM and for healthy people it is less than 0.5 pM [26,28]. The half-life of STK1p normally corresponds to the STK1p value at 1 month after radical tumor resection [25]. In this study, however, the treatment situation was complex. Before start of individualized chemotherapy, all patients with clinical early/middle stages (I–IIIA) received radical surgery, reducing the mean STK1p to a mean value of approximately 0.7 pM. Of the advanced stages (IIIB–IV), approximately 30% of patients received only local surgery, and in some cases also combined traditional Chinese medicine treatments, reducing the STK1p values to about 1.0 pM.

After two cycles of chemotherapy treatment, no decrease of STK1p was found in the early/middle (I–IIIA) group of patients (Table 2C), while the STK1p values in the patients of advance stages (IIIB–IV) decreased by 34.5% (Table 2D). The decline in the STK1p values of the early/middle stages (I–IIIA) patients was mainly due to the radical surgery, performed more than 1 month before start of the chemotherapy (Table 2A). It is important to note that in this study, approximately 70% of the patients at advanced stages (IIIB–IV) before start of the chemotherapy were unable to perform surgery and thus the STK1p values of these patients might not be reduced before the start of the chemotherapy, as found in the early-/middle-stage patients.

### Associations between low & elevated STK1p & clinical pathological characteristics after two-cycle postchemotherapy

**Figure 2.**
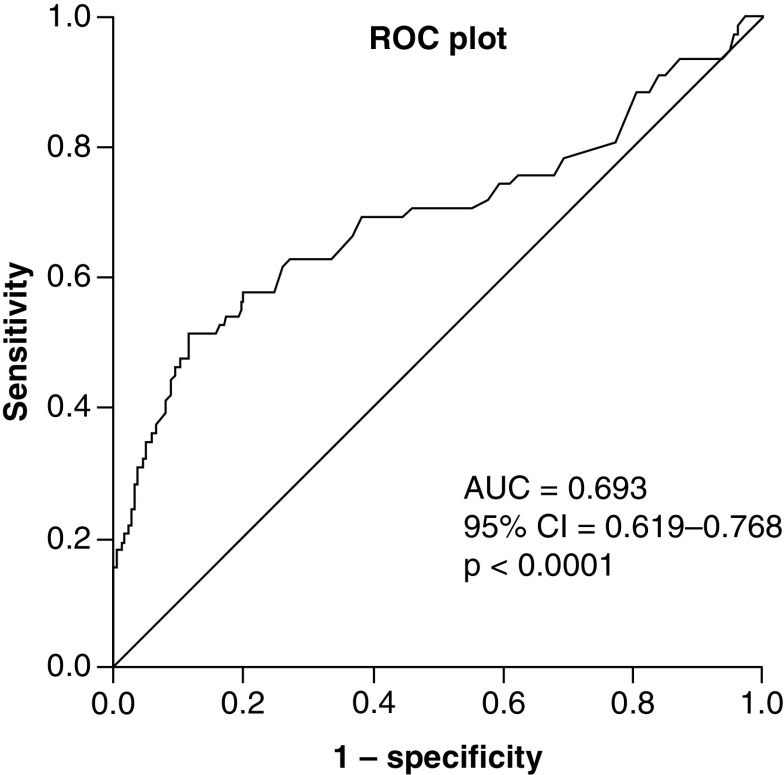
Receiver operation characteristic analysis. The analysis was based on STK1p values of 101 NSCLCs patients and 400 healthy persons. AUC: Area under the curve; NSCLC: Non-small-cell lung cancer; take away; ROC: Receiver operation characteristic; STK1p: Serum thymidine kinase 1 concentration.

To be able to identify low and elevated STK1p groups of patients, a suitable threshold level is required. This was achieved by performing an ROC statistical analysis. The ROC test of STK1p was performed between second-cycle chemotherapy patients (n = 101) and normal healthy persons (n = 400, Figure 2). The low area under the curve (AUC) value (0.693) found should be expected since the tumor patients were treated, resulting in reduced STK1 values, closer to the STK1p values of healthy persons.

The ROC analysis showed a suitable STK1p’s threshold score of 0.6 pM corresponding to a sensitivity of 0.827. Based on this threshold value, we divided the tumor patients into two group: STK1p ≤0.6 pM and STK1p >0.6 pM, as low and elevated groups of prognostic assessment, respectively. We tested patients from prechemotherapy of stage III–IV; no significant difference between low and elevated STK1p values were found (p = 0.17).

The number of patients with low and elevated STK1p values with different clinical parameters after the second cycle of chemotherapy were analyzed by Chi-square test (Table 3). The number of patients with low STK1p values were significantly higher in the early/middle stages (I–IIIA vs advanced IIIB + IV, p = 0.015), in the combined treatment group (surgery + chemotherapy vs chemotherapy alone, p = 0.010) and in the OR (PR vs PD + SD groups, p = 0.049). The higher number of patients in early stage/middle stage (I–IIIA) also correlated significantly to OS (see below). There was no difference in the number of patients of advanced stages (IIIA–IV; p = 0.529), of low STK1p value among histological type (p = 0.149), ages (p = 0.626) and sex (p = 0.054). Low and elevated STK1p groups did not significantly correlated to smoking history (p = 0.207, data not shown).

**Table 3.** Associations between the low & elevated STK1p values & clinical pathological characteristics after two-cycle chemotherapy (Chi-square test).

Group	STK1p ≤0.6 pM (n = 58)	STK1p >0.6 pM (n = 43)	p-value
Clinical stage, n = 95			
Early/middle (I–IIIA), n = 24	19	5	
Advanced (IIIB + IV), n = 71	36	35	**0.015**
Histological type, n = 98			
AC, n = 70	40	30	
SCC, n = 28	17	9	0.149
Age, n = 101			
≤60 years, n = 34	20	14	
>60 years, n = 67	36	31	0.626
Gender n = 101			
M, n = 68	40	38	
F, n = 33	17	6	0.054
RECIST, n = 101			
OR (CR + PR), n = 32	18	14	
NR (PD + SD), n = 69	31	38	**0.049**
Clinical stage: IA–IV, n = 101			
Surgery + chemo., n = 47	31	16	**0.010**
Chemo-alone, n = 54	23	31	
Clinical stage: IIIB + IV, n = 73			
Surgery + chemo., n = 23	14	9	
Chemo.-alone, n = 50	27	23	0.529
Surgery + chemo., n = 42			
Stage I–IIIA, n = 24	18	6	
Stage IIIB + IV, n = 18	8	10	**0.044**

Note: Bold values were used to indicate for the readers that these values are statistically significant.

AC: Adenocarcinoma; Chemo.: Chemotherapy; CR: Complete response; NR: No response; OR: Objective response; PD: Progressive disease; PR*:* Partial response; RECIST: Response Evaluation Criteria in Solid Tumors; SCC: Squamous cell carcinoma; SD: Stable disease; STK1p: Serum thymidine kinase 1 concentration.

### Kaplan–Meier plots assessing OS

The number who died (mortality) from our 101 NSCLC patients was 39.6%. The mortality of the patients at early/middle stages (I–IIIA) and of advanced stages (IIIB–IV) were 12.5 and 49.3%, respectively.

**Figure 3.**
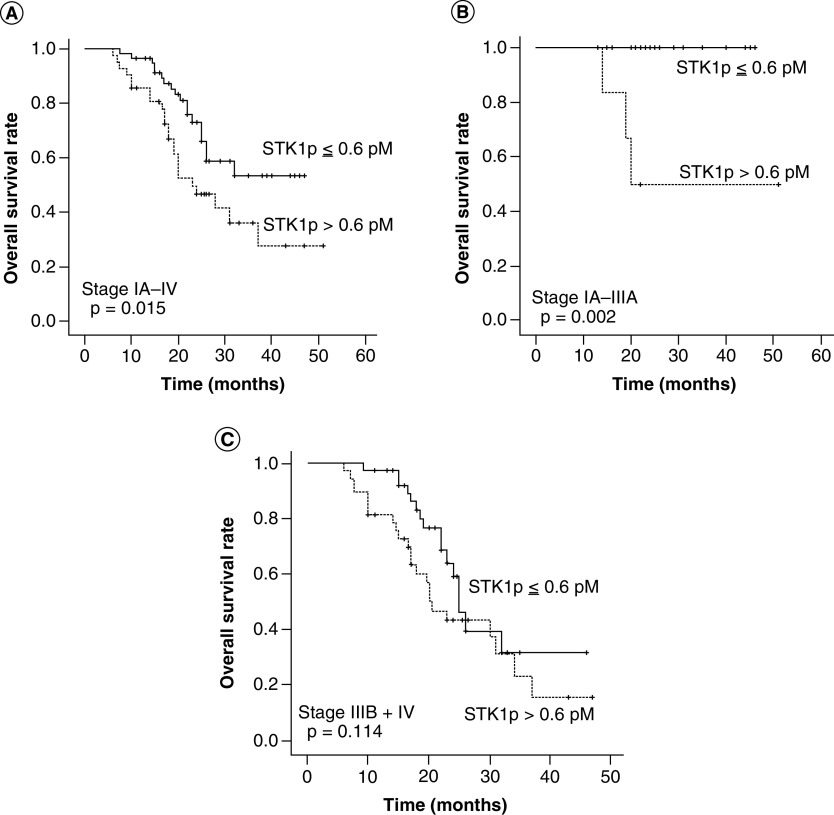
Overall survival in relation to clinical situations. The Kaplan–Meyer plots showed OS in 101 NSCLCs patients in relation to STK1p values **(A)**; OS in 24 NSCLCs patients of early/middle stages (I–IIIA) in relation to STK1p values **(B)** and 71 NSCLCs patients with advantage stages (IIIB–IV) in relation to STK1p values **(C).** Significant log rank values between the OS plots are shown in figures. The solid dots in the OS plots show the times of censored observations. NSCLC: Non-small-cell lung cancer; OS: overall survival; STK1p: Serum thymidine kinase 1 concentration.

**Figure 4.**
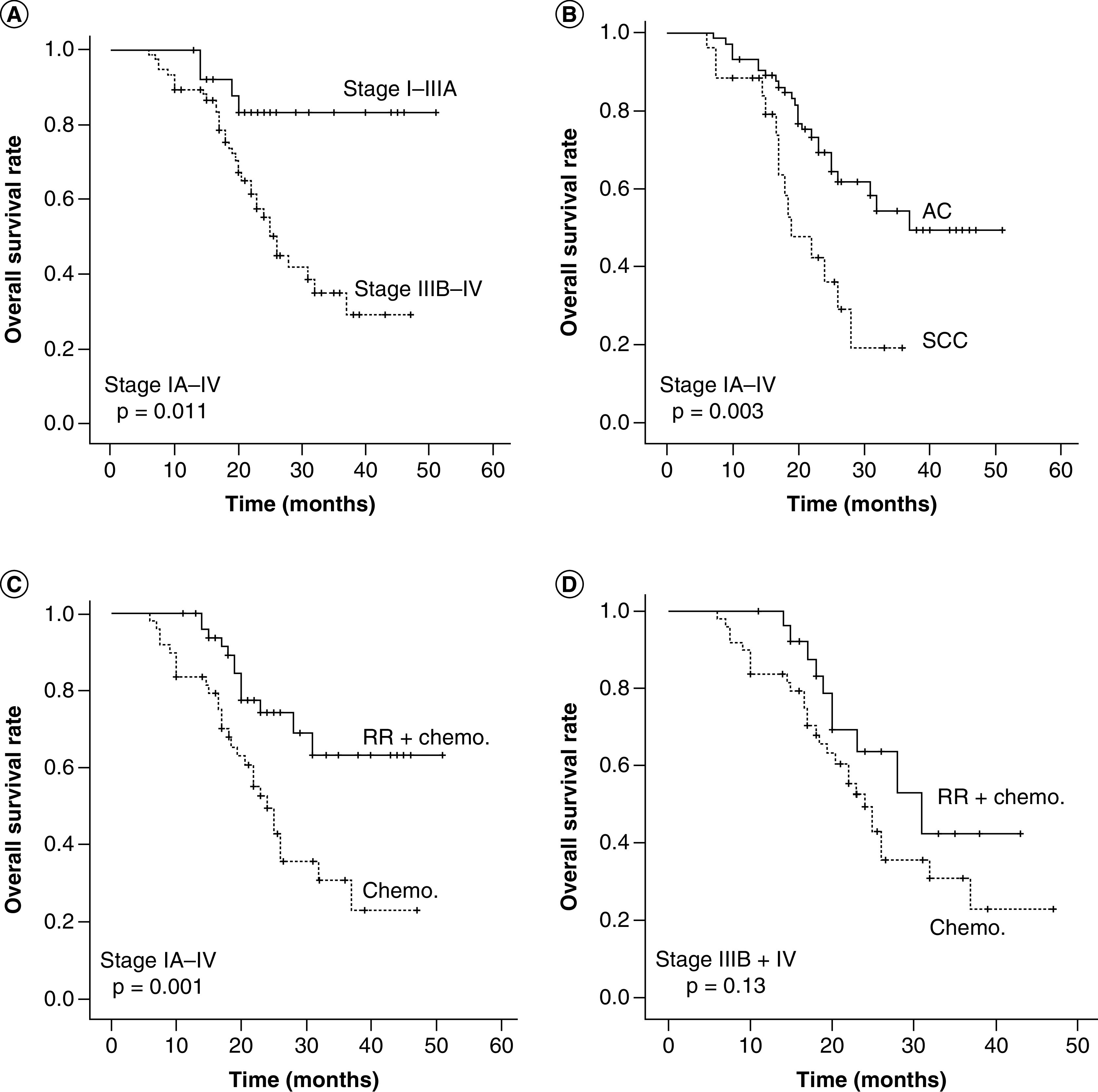
Overall survival in relation to STK1p concentration. The Kaplan–Meyer plots showed OS in 95 NSCLCs patients with stages IA–IV **(A)**; 98 histological type **(B)** and 101 NSCLCs patients after treatment (surgery + chemo. vs chemo.-alone) **(C)** and 71 NSCLCs patients with advantage stages IIIB–IV **(D).** Significant log rank values between the OS plots are shown in the figures. The solid dots in the OS plots show the times of censored observations. AC: Adenocarcinoma; Chemo.: Chemotherapy; NSCLC: Non-small-cell lung cancer; OS: overall survival; RR: Radical resection; SCC: Squamous cell carcinoma.

In Figure 3, we compare the OS at low and high STK1p values and in Figure 4 the OS of different clinical stages, different pathological types and treatment regimes. The Kaplan–Meier OS plots of low and elevated STK1p values are shown for total stages (IA–IV, Figure 3A), early/middle stages (IA–IIIA, Figure 3B) and for advanced stages (IIIB–IV, Figure 3C).

Patients with elevated STK1p values were associated with poor OS (stages IA–IV, p = 0.015, Figure 3A), especially for patients with early/middle stages (IA–IIIA, p = 0.001, Figure 3B). In patients with advanced stages (IIIB–IV), there was a tendency of improved OS at low STK1p values during the early follow-up time (about 0–20 months), but when regarded over the total follow-up time, no significant difference was found (p = 0.114, Figure 3C). There was an improved OS in patients with early/middle stages (IA–IIIA) compared with advanced stages (IIIA–IV) (p = 0.011, Figure 4A) and in AC patients compared with SCC patients (p = 0.003, Figure 4B). There was also an improved OS of patients (I–IV) receiving combined treatment (surgery + chemotherapy) compared with chemotherapy alone (p = 0.001, Figure 4C). Patients with advanced stages (IIIB–IV) had no benefit from combine treatment (surgery + chemotherapy, p = 0.13, Figure 4D).

There was no significance difference in OS with age (p = 0.132), sex (p = 0.097) or chemotherapy with NR (p = 0.432, data not shown).

### Uni- & multi-variate Cox regression analyses for OS

The uni- and multi-variate COX analysis of STK1p, clinical stages, treatment options, histological types and sex are shown in Table 4.

**Table 4.** Uni- and multi-variate COX analysis of STK1p, clinical stage, treatment options, histological type and sex.

Univariate	p-value	Hazard risk	95% CI
Surgery + chemo. vs chemo.-alone	**0.002**	2.763	1.431–5.342
STK1p (≤0.6 vs >0.6 pM)	**0.018**	2.108	1.137–3.909
Stage (I–IIIA vs IIIB–IV)	**0.008**	4.044	1.437–11.38
Histological type (AC vs SCC)	**0.004**	2.538	1.355–4.294
Gender (M vs F)	0.099	1.976	1.042–3.744
Multivariate	p-value	Hazard risk	95% CI
Surgery + chemo. vs chemo.-alone	**0.001**	3.04	1.543–5.987
STK1p (≤0.6 vs >0.6 pM)	**0.010**	2.295	1.222–4.308
Stage (I–IIIA vs IIIB–IV)	0.385		
Histological type (AC vs SCC)	0.276		
Gender (M vs F)	0.293		

AC: Adenocarcinoma; Chemo.: Chemotherapy; F: Female; M: Male; SCC: Squamous cell carcinoma; STK1p: Serum thymidine kinase 1 concentration.

Although mortality and OS are important parameters, COX regression values are most important when judging if a prognostic marker is independent or not. The univariate analysis showed prognostic significance of STK1p expression (p = 0.018), clinical stages (p = 0.008), histological types (p = 0.004) and treatment regimen (p = 0.002), but not for sex (p = 0.099), ages (p = 0.596) and chemotherapy with NR (p = 0.729, data not shown).

The multi-variate analysis showed that the STK1p expression (p = 0.01) and treatment regimen (p = 0.001) were independent prognostic factors for OS, while stages, histological types and sex were not (p > 0.05). A case analysis describing those who died at early stages is provided in the Supplementary material.

## Discussion

The purpose of this study was to find out if STK1p could give any information about the efficacy of treatment of NSCLC patients, useful for decisions regarding additional treatments. This observational study was based on the RWD concept of individual adapted treatment. The novelty in this study is that we used STK1p, a biomarker closely related to tumor proliferation. Most of the prognostic biomarkers in use today are related to the size/volume of the tumor, which does not necessarily mean high proliferation rates. The end points were OR, OS and mortality; treatment regime, clinical stages, pathological types, ages and genders were also included.

First, based on the actual data after two cycles of individualized chemotherapy, we divided the patients into two groups of low (≤0.6 pM) and high (≥0.6 pM) STK1p value, creating an optimal cut-off STK1p value using ROC analysis (0.6 pM). These two groups were than compared with clinical parameters. The results are shown in Figures 3 & 4 and in Tables 2–4. Although the patients were treated with several cycles, we found that the second cycle of the chemotherapy was optimal when comparing STK1p with the clinical parameter of OR (PR vs PD + SD, p = 0.049, Table 3). This is in agreement with previous studies, for example, in NSCLC [29] and breast carcinoma [33].

We found that STK1p level of treated early/middle stages (I–IIIA) NSCLC patients correlated to mortality and OS, but not to patients at advanced stages (IIIb-IV). The number of patients with low and elevated STK1p values were compared with clinical parameters after second-cycle chemotherapy, reflecting different physiological states of the tumor (analyzed by Chi-square test, Table 3). We found that patients with low STK1p values correlated significantly to early/middle stages (I–IIIA, before chemotherapy), to patients after receiving combined treatment (surgery + chemotherapy, p = 0.015). The low STK1p score was significantly correlated with OR (p = 0.049) indicating that STK1p reflects different growing states of the tumor and effects of the surgery + chemotherapy response (Table 3). The OS also showed that elevated STK1p values were associated with poor OS (p = 0.015, Figure 3A), especially in early/middle stages (IA–IIIA, p = 0.002, Figure 3B). However, the OS was not able to distinguish between low and high STK1p groups of patients with advantage stages (IIIA–IV) (p = 0.114, Figure 3C). It has been reported that the STK1p expression correlates with tumor proliferation in early/middle stages (I–IIIA) of NSCLCs, but not in advanced stages (IIIB–IV) [19,34]. In addition, and not related to STK1p, poor OS was found in patients of advanced stages IIIB–IV (p = 0.011, Figure 4A), in patients of pathological type SCC (p = 0.003, Figure 4B) and after chemotherapy alone (p = 0.001, Figure 4C), but not in early/middle (I–IIIA) patients receiving surgery + chemotherapy. However, it seems that the OS rate of advanced stage (IIIB + IV) patients may be slightly improved in patients with low STK1p values before start of the chemotherapy (p = 0.114, Figure 3C). However, this was not confirmed by the mortality, which was still high after treatments (49.3%). We also tested patients from prechemotherapy treatment on patients with stage III–IV; no significant difference with low and elevated STK1p values was found (p = 0.17, data not shown).

In addition, in a previous study on primary (M0) and metastatic (M1) renal cell carcinoma (RCC) patients, immunohistochemistry showed that the density and intensity of STK1p expression in RCC tissue were positively correlated to tumor diameters up to 7 cm, but inversely related to tumors size above 7 cm, the latter due to increasing necrosis [35]. Thus, it should be noted that STK1p is may not a suitable prognostic marker when there is a risk of extensive necrosis, at least in advanced stage of NSCLC patients. Therefore, we concluded that the best outcome of using STK1p is in the early/middle stages of NSCLC [29,33]. This is in agreement with a previous study where it was proposed that STK1p can be used for monitoring treatment response to individualized therapy at early tumor stages, as well as in developing new cytotoxic drugs or targeted therapies [33].

We found that the mean STK1p value after the second cycle of chemotherapy was still significantly higher (0.566 pM) than in healthy controls (p = 0.38 pM), characterized as NR of the treatment (Table 2). This indicates that some residual tumors were still present (metastasis), regardless of treatment and correlated with elevated STK1p values. In the cases reported (see the Supplementary material), imaging examination confirmed this, showing that some patients were already in a metastatic stage before start of the chemotherapy. The average value of STK1p in healthy people has been reported in a range of 0.31–0.39 pM, based on a meta-analysis of healthy screening of 160,086 people [28].

Although OS and mortality show that STK1p levels are a prognostic factor, COX regression analysis is important to perform when trying to judge if a marker is independent or not. The multi-variate COX regression analysis showed that STK1p (p = 0.01) was a significant independent prognostic factor for OS. To confirm this conclusion, the number of patients needs to be expanded in future studies. However, we propose that STK1p can be used as a reasonable individual prognostic factor, particularly for patients of early/middle stages (I–IIIB), for improved prediction of the outcome of the treatment.

Recently, the immunotherapy revolution, specifically development of immune checkpoint inhibitors, has improve the treatment of advanced lung cancer [36,37]. A meta-analysis revealed that immunotherapy in combination with chemotherapy is an effective option as a first-line treatment for lung cancer (n = 4887) [38]. NSCLC patients administered with immunotherapies exhibited better progression-free survival and OS than patients treated with chemotherapy alone (p < 0.001). Moreover, as the expression of PD-L1 increased, the progression-free survival and OS were more significant [39]. We suggest that STK1p assay could be used for the assessment of the effects of immunotherapy individually.

TK1 is a precise molecule for monitoring and prognosis, specifically, for early detection of invisible small tumors. The STK1p assay we used in this study is also noninvasive and of low cost [22]. We propose that STK1p should be combined with imaging, other biomarkers (e.g., CYFRA21-1, CEA and CTCs) and TK1 immunohistochemically staining, to provide a reliable method for assessment of the development of premalignancy or diseases associated with the risk process of lung cancer. There would be limited lung cancer development in the future if we could discovery premalignancy, or treat early tumors in time.

## Conclusion

Although this study is nonrandomized using an RWD concept, it shows that low STK1p value correlated significantly to favorable survival in early-/middle-stage (I–IIIA) NSCLC patients. STK1p also identifies patients with good survival after combined treatment of surgery + chemotherapy. The COX multi-variate analysis shows that STK1p is an independent prognostic biomarker for OS.

STK1p is a valuable biomarker at low cost. It may be useful as a tool for monitoring treatment efficacy and evaluating the prognosis of treatment for individual patients at early/middle stages of NSCLC.

## Future perspective

TK1 is a useful biomarker for cell proliferation and has been suggested to be used for evaluating tumor proliferation in oncology for more than 40 years. A number of publications have demonstrated that TK1 is a potential biomarker for prediction of survival and recurrence, and monitoring of tumor treatment effect, both in serological assay and immunohistochemical staining. Although we have improved serum TK1 (STK1) assays based on TK activity and TK1 antibodies, and extensive publications, the STK1 evidence is thus far not enough to be able to be recommend use of TK in serum. Therefore, clinical trials or RWD investigations on TK are now being performed and will likely resulting in a recommendation of STK1p as a biomarker in oncology soon.

Extensive oncology clinical studies on TK1 have been performed in China during recent years, based on specific chicken antihuman TK1 IgY poly-antibody against TK1 in serum (STK1p). More than 300 oncology clinics and health screening centers are now involved in evaluating the usefulness of STK1p, with more than 400 basic and clinical publications thus far.

The development of TK1 antibodies made it possible to expand the use of serum TK1 from lymphoma and leukemia, mainly based on TK activity in serum, to almost all type of solid human tumors. This further increased the interest in STK1p as a tumor proliferating biomarker in human oncology.

A useful tumor-related biomarker requires a simple and fast assay. The STK1p methods used today are still time and resource consuming. However, a TK1 kit adjusted for an automatic chemiluminescence analyzer based on magnetic beads has been developed, which realized the effectiveness of the TK1-immune sandwich technology for routine health screening (www.sstkbiotech.com). The TK1 assay time is reduced to about 1 h and the coefficient of variations (CV) to less than 5%. This TK1 kit will be licensed for clinical use in early 2021.

Although STK1p shows good correlations to clinical parameters, combination with other tumor-related biomarkers is to be recommended. We recommend the use of STK1p together with immunohistochemistry of TK1 and other tumor-related makers.

**Summary points**

- Lung cancer is one of the most common types of tumors with a high mortality rate.
- We investigated if thymidine kinase 1 in serum (STK1p) is a reliable prognostic marker for survival for non-small-cell lung carcinoma (NSCLC).
- The STK1p values of 127 patients were determined by an enhanced chemiluminescent dot blot assay.
- Patients with elevated STK1p values had worse overall survival (OS), especially patients of early/middle stages.
- Multi-variable analysis showed that STK1p value and combined treatment surgery + chemotherapy were independent prognostic factors for favorable OS.
- STK1p predicts OS of early-/middle-stage (I–IIIA) NSCLCs patients following a nonrandomized individual adapted treatment, but it may be not useful in advanced stages (IIIB + IV) of NSCLCs.
