# Supplementary material for: STK1p as a prognostic biomarker for overall survival in non-small-cell lung carcinoma, based on real-world data
Source: Future Sci OA. 2020 Nov 23;7(3):FSO661. doi: 10.2144/fsoa-2020-0130 (PMC7849927; doi:10.2144/fsoa-2020-0130)
Supplement: Supplementary file 1 [file fsoa-07-661-s1.docx]

**Supplemental material**

**Cases analysis of early stage dead patients**

Although the patients of early stages (I-IIIA) showed a good prognosis with high OS rates (Table 1, figure 1B), it was noted that 3 patients of stage IIA-IIB died within 14-20 months after end of the treatment. We observed that the STK1p values after 2-cycle individual-chemotherapy did not returned to normal values (<0.6 pM), characterized as NR (no response, SD) by CT examination. The main reason for the death might be development of metastasis. Here we report a case analysis of these three patients to prove the significant correlations between STK1p values and clinical data in this RWD study.

**Case 1.** A 65 years old woman with a mucinous adenocarcinoma, clinical stage IIA, grade G2 and a tumour size of 4.0*2.3 cm. She was found to have right middle lung irregularity after operation. In 2016-01-26 she was found to have multiple small nodules in both lungs by CT observation, and was considered for metastasis. She immediately started chemotherapy with pemetrexed disodium+ carboplatin for 4 cycles from 2016-01-27 to 2016-05-04. The CT re-examination in 2016-04-01 showed multiple small asymptomatic malignant lung nodules in both lungs, similar to the observation in 2016-01-26. Therefore, she continued to treatment with docetaxe from 2016-05-04 to 2016-08-04. She was re-examined by CT in 2016-07-06 and in 2016-12-26, showing that the multiple small asymptomatic malignant lung nodules were still presence in both lungs and slightly larger than found in 2016-07-06, and considered for metastasis. She dyed 20 months after the treatment. The STK1p pre-chemotherapy value was 0.27 pM and after 2-cycle-post-chemothyrapy increased to 0.82 pM, increasing 3.5-fold times compared to the base line. The chemotherapy showed SD.

**Case 2**. Man, 78 years. He was found to have a right upper lung tumour and did radical resection of right upper lung with thoracoscopy under general anesthesia in 2014-05-29, and conformed adenocarcinoma (clinical stage IIA, grade G3 and tumour size of 5.5 cm). He performed a maintenance therapy with pemetrexed disodium + carboplatin for 4 cycles in 2015-06-09 to 2015-09-22. Abnormal changes were found in the right lung, bilateral pleural effusion (partially encapsulated), and fibrous foci in left upper lung, by CT detection in 2015-07-12. It was also found liver cysts and left lateral adrenal branch hyperplasia. He dead 14 months after start of the chemotherapy. The STK1p value pre-chemotherapy was 0.1 pM and 2-cycle-post-chemothyrapy increased to 5.29 pM, an about 60-fold increase compared to the base line. The chemotherapy showed SD.

**Case 3.** Man, 78 years. He was found to have a left upper lobe lung tumour and conformed adenocarcinoma, clinical stage IB, grade G3 and a tumour size of 3.5*3.0*1.5 cm. He performed a neo-chemotherapy with doxorubicin + nedaplatin for 6 cycles in 2013-05-21 to 2013-10-22. The right interstitial inflammation improved slightly, but no nodules in left upper lobe were seen by CT in 2013-12-30. He did radical resection in 2014-04-17 and then continued chemotherapy. The chemotherapy planed 1) Doxorubicin + carboplatin for 3 cycles in 2014-11-26 to 2015-01-27; 2) Gemcitabine for 2 cycles in 2015-03-03 to 2015-04-03; 3) Gemcitabine + nedaplatin for 2 cycles in 2016-03-26 to 2016-05-03; 4) Vinorelbine + nedaplatin for 2 cycles in 2016-06-01 to 2016-07-23.

The PET-CT detection in 2015-02-26 after surgery and chemotherapy showed that the third and fourth posterior ribs were destroyed and local pleura was thickened as well as accompanied by increased glucose metabolism. He also considered to have metastasis. He was re-examined with CT in 2016-03-10 and suggested to have multiple asymptomatic malignant lung nodules, multiple right ribs and thoracic vertebrae metastasis. He dead 15 months after start of the chemotherapy treatment. The STK1p value pre-chemotherapy was 0.88 pM and after 2 cycle-post-chemotherapy 0.93 pM, means that the STK1p did not changes to compared to the base line. The chemotherapy showed SD.
